# Demographic and Socioeconomic Disparities in Adolescent Obesity: Insights From the National Survey of Children’s Health Database

**DOI:** 10.7759/cureus.72150

**Published:** 2024-10-22

**Authors:** Oluwatosin O Arubuolawe, Oluwadamilare T Gabriel, Chioma J Anats, Lilian O Odion-Omonhimin, Paul A Momodu, Saidat A Akanbi, Rauf B Babilsie, Kalpana Giri, Okelue E Okobi

**Affiliations:** 1 Psychiatry and Behavioral Sciences, Manhattan Psychiatric Center, New York, USA; 2 Pediatrics, Hopkins Center for Rehabilitation &amp; Healthcare, New York, USA; 3 Pediatrics, Maimonides Medical Center, Brooklyn, USA; 4 Medicine and Surgery, University of Benin, Benin, NGA; 5 Medicine, International University of the Health Sciences, Basseterre, KNA; 6 School of Public Health, Boston University, Boston, USA; 7 Internal Medicine, Asesewa Government Hospital, Asesewa, GHA; 8 Family Medicine, Zainul Haque Sikder Women's Medical College &amp; Hospital, Dhaka, BGD; 9 Family Medicine, Medficient Health Systems, Laurel, USA; 10 Family Medicine, Lakeside Medical Center, Belle Glade, USA; 11 Family Medicine, Larkin Community Hospital Palm Springs Campus, Miami, USA

**Keywords:** adolescent obesity, adverse childhood experiences, bmi percentile, income, insurance status, national survey of children’s health, racial disparities, socioeconomic factors

## Abstract

Background: Obesity among adolescents is a significant public health concern. This study analyzes the prevalence of obesity among adolescents using data from the National Survey of Children’s Health (NSCH). The analysis focuses on the impact of race and socioeconomic variables on obesity rates.

Objective: This study aimed to examine how race and socioeconomic factors, including income level, insurance status, and adverse childhood experiences, influence obesity prevalence among adolescents.

Methods: Data were extracted from the NSCH for adolescents aged 10-17 years, defining obesity as a body mass index (BMI) of or above the 95th percentile (n = 3,344). Furthermore, for this study, the researchers utilized SPSS version 26 software for data analysis. The analysis focused on obesity prevalence by race (Hispanic, Black non-Hispanic, and White non-Hispanic) and socioeconomic factors, including family income relative to the federal poverty level (FPL), insurance status, and adverse childhood experiences. Obesity was assessed by reporting prevalence estimates and confidence intervals (CIs).

Results: Among adolescents aged 10-17 years (n = 23,452 ), the overall prevalence of obesity was 16.6% (CI: 15.6%-17.7%), overweight 15.2% (CI: 14.3-16.1%), normal weight 60.9% (CI: 59.6%-62.2%), and underweight 7.3% (CI: 6.5%-8.1%). Stating that the overall *n* at the beginning helps the reader to assess the study population and the percentages are more impactful in communicating the severity of the problem or outcome. We found significant disparities in obesity prevalence among different racial and socioeconomic groups. Racial disparities in obesity prevalence were evident, with 773 Hispanic adolescents having the highest obesity prevalence at 21.9%, followed by 330 Black non-Hispanic adolescents at 21.3%. Socioeconomic status was inversely related to obesity prevalence; among 647 adolescents from households with income at 0-99% of the FPL, the obesity prevalence was 22.5%, while 1,630 had a normal weight prevalence of 53.2%. For 3,150 insured adolescents, the obesity prevalence was 16.6%, and 14,481 had a normal weight prevalence of 61.3%. A total of 1,121 adolescents with two or more adverse childhood experiences (ACEs) had a 22.4% obesity prevalence.

Conclusion: The study highlights significant racial and socioeconomic disparities in adolescent obesity rates. Higher obesity prevalence is associated with lower income levels, lack of insurance, and adverse childhood experiences. These findings suggest the need for targeted interventions addressing these disparities to improve adolescent health outcomes. The targeted interventions are essential, as they will aid in addressing the observed disparities by tackling factors contributing to obesity within the demographic group. Through the execution of the targeted interventions, we can customize resources and strategies to the adolescent population's needs, ascertaining an increasingly equitable approach to lowering obesity prevalence rates and enhancing the health outcomes in adolescents.

## Introduction

Adolescent obesity represents a critical public health challenge in the United States, carrying profound implications for the future well-being of the population [1]. Over the past few decades, the increasing prevalence of obesity among adolescents aged 10-17 years has sparked significant concern due to its potential long-term health consequences [1,2]. Adolescents who are obese are at a heightened risk of developing serious health conditions such as type 2 diabetes, hypertension (HTN), cardiovascular disease (CVD), and psychological disorders, many of which can persist and worsen into adulthood [2,3]. Moreover, adolescent obesity represents an important public health challenge in the United States and across the globe, as it carries weighty implications with regard to the future well-being of the global population. In this regard, attention has been accorded to individuals aged 0-17 years, especially adolescents, owing to the extraordinary health considerations that tend to emerge during such a developmental period. Thus, individuals aged 12-17 years have been found to be at a relatively high risk of developing obesity, which has further been associated with different chronic long-term health outcomes that include type 2 diabetes, HTN, CVDs, and psychological disorders [2-4]. Furthermore, various studies have stressed the significance of studies focusing on adolescents owing to their fast psychological/emotional, social, and physical changes that affect both their health behaviors and outcomes not only during adolescence but also in adulthood [3-6]. Still, the obesity prevalence disparities across different demographic groups have been stressed by various studies, indicating that adolescent obesity effects are unevenly distributed with racial and socioeconomic aspects playing important roles in the determination of the risk levels [4,5].

Addressing this growing issue necessitates a comprehensive understanding of the contributing factors and the disparities that exist across various demographic groups [1-4]. Recent data reveal that the prevalence of obesity among United States adolescents is alarmingly high, with approximately 21% of those aged 10-17 years classified as obese [1,3,4]. This trend is particularly concerning given the associated health risks that often extend into adulthood, including an increased likelihood of developing cardiovascular disease and type 2 diabetes [3,6]. While a range of treatment options is available, including lifestyle modifications, pharmacotherapy, and surgical interventions, these treatments have limitations. For example, bariatric surgery, which can lead to a significant body mass reduction (BMI) reduction of ~30%, is generally reserved for severe cases but does not offer a comprehensive solution for the broader adolescent population [3,6,7].

The pathophysiology of adolescent obesity is complex and multifactorial, involving an interplay of genetic, environmental, and socioeconomic factors. Fundamentally, obesity results from an energy imbalance where caloric intake surpasses energy expenditure, leading to an excessive accumulation of fat [8]. While genetic predispositions can influence metabolism and fat storage, environmental and socioeconomic factors play a particularly significant role. Adolescents from lower socioeconomic backgrounds often face barriers such as limited access to nutritious foods and safe spaces for physical activity, both of which contribute to unhealthy weight gain [8-10]. In addition, chronic stress stemming from economic hardship and social inequities can trigger hormonal changes, such as elevated cortisol levels, which promote fat storage. Other contributing factors include racial and ethnic disparities in dietary habits, limited healthcare access, and systemic inequalities [1,2,8-10]. The objective of this study was to analyze data from the National Survey of Children’s Health (NSCH) to determine the prevalence of obesity in adolescents aged 10-17 years. 

## Materials and methods

Study design and data source

For this retrospective study, we utilized data from the NSCH, a nationally representative survey conducted annually by the United States Census Bureau on behalf of the Health Resources and Services Administration’s Maternal and Child Health Bureau, to explore the scope and underlying factors of adolescent obesity. The NSCH collects extensive data on the health, well-being, and demographic characteristics of children in the United States, including BMI for adolescents aged 10-17 years [11]. The NSCH also provides a comprehensive dataset that allows for an in-depth examination of obesity prevalence across the United States, with a particular focus on how demographic and socioeconomic factors influence these rates [11]. The inclusion criteria required complete data on BMI, race, income, insurance status, and exposure to adverse childhood experiences (ACEs). Adolescents with missing information on any of these variables were excluded from the analysis to maintain the integrity of the dataset and ensure accurate results.

Outcome measure and variables

The primary outcome measure was the prevalence of obesity among adolescents, operationally defined as a BMI at or above the 95th percentile for age and gender, according to the growth charts provided by the Centers for Disease Control and Prevention (CDC). BMI was calculated using height and weight data reported by parents or guardians as part of the NSCH survey. Moreover, for this study, BMI was mainly categorized into four groups, namely, underweight (BMI < 18.5), normal weight (BMI 18.5-24.9), overweight (BMI 25.0-29.9), and obese (BMI ≥ 30.0). The analysis stratified the outcome by race, income level, insurance status, and ACEs to examine disparities across these socioeconomic variables. Thus, the stratification of BMI by race by the analysis was mainly to evaluate the potential disparities across divergent racial groups.

In addition, we evaluated the existing correlations between obesity and various socioeconomic factors that included family/household income, insurance status, and ACEs. The family/household income was classified into four groups on the basis of FPL: 0-99% FPL, 100-199% FPL, 200-399% FPL, and 400% FPL and above. The insurance status was further categorized into two groups: insured and uninsured. Still, the ACEs were categorized into three main groups: no ACEs, one ACE, and two or more ACEs. The variables were subsequently evaluated for their potential correlation with obesity in adolescents.

Data analysis

Descriptive statistics were used to summarize the characteristics of the study population. The prevalence of obesity was calculated for the entire sample and stratified by race, income level, insurance status, and ACEs. The statistical analyses were mainly conducted through the use of IBM SPSS Statistics for Windows, Version 26.0 (released 2019, IBM Corp., Armonk, NY). Moreover, for all the statistical tests, the alpha threshold of significance was set at 0.05. Moreover, 95% confidence intervals (CIs) were computed to assess the precision of prevalence estimates. Chi-square tests were employed to compare obesity prevalence rates across different demographic and socioeconomic groups. All analyses were conducted using survey weights provided in the NSCH dataset, which account for the complex sampling design, ensuring the results are nationally representative. Statistical analyses were performed using 95% CIs, in the assessment of the precision of prevalence estimates.

Ethical considerations

The NSCH data are publicly available and de-identified, ensuring participant confidentiality. Since this study involved secondary analysis of publicly available data, it does not require institutional review board (IRB) approval.

## Results

The analysis of adolescent obesity among individuals aged 10-17 years highlights significant prevalence rates across various demographic factors, including gender, age, race, and socioeconomic status. The study found that, among the adolescents, 3,342 individuals had obesity, representing an obesity rate of 16.6% (CI: 15.6-17.7%), even though there were notable variations across different groups. When examining weight categories, most adolescents fell within the normal weight range (BMI 5th to 84th percentiles), as 15,148 adolescents fell within the range, representing a prevalence of 60.9% (CI: 59.6-62.2%). However, a considerable portion of the population, 1,560 individuals, also fell into the categories of underweight, representing 7.3% (CI: 6.5-8.1%), while another 3,408 were overweight, representing 15.2% (CI: 14.3-16.1%). Table 1 presents the national outcome measure for demographic variables. These percentages indicate that while a majority of adolescents maintain a healthy weight, a significant minority struggle with weight issues on both ends of the spectrum.

**Table 1:** National outcome measure for demographic variables: percentage of adolescents (age 10-17 years) CI: confidence interval, %: percentage, Pop. Est.: population estimates, (n): number of participants/sample population

Variables	Total national data				Based on gender							Based on age						
	Overall				Male			Female			P value	10-13 years old			14-17 years old			P-value
Characteristics	%	CI	Sample count	Pop. Est.	%	CI	Sample Count	%	CI	Sample Count	-	%	CI	Sample Count	%	CI	Sample Count	-
Underweight (less than 5th percentile)	7.3	6.5 - 8.1	1,560	2,420,144	7.9	6.8 - 9.1	901	6.7	5.6 - 7.9	659	p-value < 0.05	7.6	6.6 - 8.8	789	6.9	5.8 - 8.2	771	p-value = 0.04
Normal weight (5th to 84th percentile)	60.9	59.6 - 62.2	15,148	20,263,238	56.9	55.1 - 58.7	7,386	65.1	63.3 - 66.9	7,762	p-value < 0.05	57.5	55.6 - 59.4	6,118	64.3	62.5 - 66.0	9,030	p-value = 0.04
Overweight (85th to 94th percentile)	15.2	14.3 - 16.1	3,408	5,052,795	15.7	14.5 - 17.0	1,832	14.7	13.3 - 16.1	1,576	p-value < 0.05	16.4	15.0 - 17.8	1,672	14	12.9 - 15.3	1,736	p-value = 0.04
Obese (95th percentile or above)	16.6	15.6 - 17.7	3,342	5,523,184	19.5	18.0 - 21.1	2,041	13.6	12.4 - 14.9	1,301	p-value < 0.05	18.5	16.9 - 20.1	1,622	14.8	13.5 - 16.1	1,720	p-value = 0.04

Gender-based analysis

The data reveal a marked difference in obesity rates between male and female adolescents. Among males, 2,041 individuals, representing 19.5% (CI: 18.0-21.1%) of males were classified as obese, in comparison to 1,301 females representing 13.6% (CI: 12.4-14.9%) of female participants. In addition, 1,832 males representing 15.7% (CI: 14.5-17.0%) were overweight, compared to 1,576 females representing 14.7% (CI: 13.3-16.1%). While the prevalence of overweight is somewhat comparable between genders, the gap in obesity rates is significant. Moreover, regarding normal weight, 7,762 females had a higher prevalence at 65.1% (CI: 63.3-66.9%) compared to 7,386 males who had a higher prevalence at 56.9% (CI: 55.1-58.7%). Conversely, 901 males presented a slightly higher underweight rates at 7.9% (CI: 6.8-9.1%) compared to 659 females at 6.7% (CI: 5.6-7.9%). The chi-square tests (p-value < 0.05) showed significant differences in obesity prevalence across the gender group. 

Age-based analysis

Obesity prevalence also varied significantly across different age groups. A total of 1,622 adolescents aged 10-13 years exhibited a higher obesity prevalence of 18.5% (CI: 16.9-20.1%) compared to 1,720 adolescents aged 14-17 years, who had an obesity rate of 14.8% (CI: 13.5-16.1%). In addition, the prevalence of overweight was higher in the younger age group as 1,672 individuals representing 16.4% (CI: 15.0-17.8%) were overweight compared to the 1736 individuals in the older group, representing 14.0% (CI: 12.9-15.3%). The underweight prevalence was also slightly higher among the younger adolescents with 789 individuals representing 7.6% (CI: 6.6-8.8%) compared to 771 individuals in the older group, representing 6.9% (CI: 5.8-8.2%). The p-value = 0.04 showed significant differences in obesity prevalence across the age groups. These findings highlight the need for targeted interventions at younger ages to prevent obesity and its associated health risks.

Racial disparities in obesity

**Figure 1:**
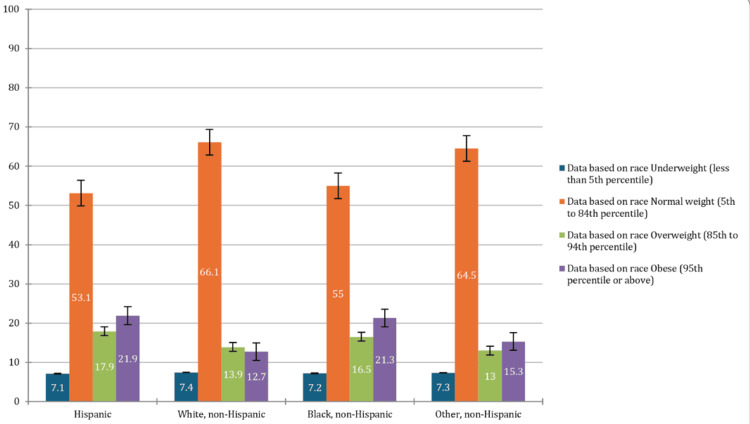
Chart comparing the weight group distributions (based on the body mass index (BMI)) among adolescents of different racial categories X-axis: percentage of adolescents, Y-axis: race/ethnicity

Racial disparities in obesity prevalence were also prominent. Thus, 773 Hispanic adolescents had the highest obesity rate at 21.9% (CI: 19.3-24.7%), closely followed by 330 Black non-Hispanic adolescents at 21.3% (CI: 18.0-25.1%). These rates are significantly higher compared to White non-Hispanic adolescents, as 1,834 of them had an obesity rate of 12.7% (CI: 11.7-13.6%). The White non-Hispanic group also had the highest normal weight prevalence with 10,144 individuals, representing 66.1% (CI: 64.8-67.4%), indicating a notable difference in weight outcomes based on race. Still, 405 non-Hispanic adolescents had an obesity rate of 15.3% (CI: 13.2-17.6%), while 2,068 of them had a normal weight prevalence of 64.5% (CI: 61.5-67.4%). These figures underscore the significant racial and ethnic disparities in obesity rates, with minority groups, particularly Hispanic and Black non-Hispanic adolescents, being disproportionately affected. The p-value < 0.05 showed significant differences in obesity prevalence across the racial category. The racial category percentage of adolescents based on BMI percentile is presented in Figure 1.

Socioeconomic factors

**Figure 2:**
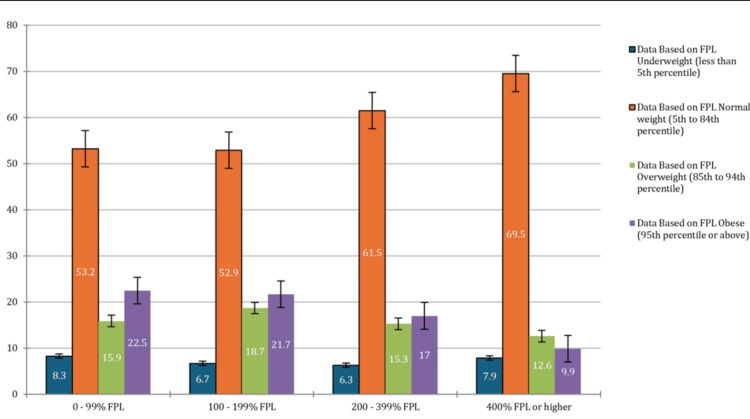
Family income category percent of adolescents based on the body mass index (BMI) percentile FPL: federal poverty level, X-axis: FPL (federal poverty level), Y-axis: percentage of adolescents

The analysis disclosed considerable socioeconomic disparities with regard to the prevalence of obesity among adolescents. For instance, adolescents from households with income levels that ranged between 0% and 99% of the federal poverty level (FPL) presented the highest obesity prevalence at 22.5% (CI: 19.9-25.3%), in comparison to the normal weight prevalence of 53.2% (CI: 49.5-56.8%). Furthermore, the adolescents within this income category presented an underweight prevalence of 8.3% (CI: 5.8-11.9%). Nevertheless, for adolescents from households within incomes that ranged between 100% and 199% of the FPL, an obesity prevalence of 21.7% (CI: 19.0-24.7%) was reported, alongside an underweight prevalence of 6.7% (CI: 5.0-8.9%). On the contrary, for the adolescents from the higher-income households of 400% FPL or higher, a considerably lower obesity rate of 9.9% (CI: 8.8-11.2%) presented alongside the highest normal weight prevalence of 69.5% (CI: 67.9-71.1%). The observed differences with regard to obesity prevalence across the different income categories were statistically significant (p < 0.05), which underscores the sturdy correlation between higher obesity prevalence and lower socioeconomic status among adolescents. The family income category percentage of adolescents based on the BMI percentile is presented in Figure 2.

Insurance and adverse childhood experiences

Insurance coverage also plays a critical role in obesity prevalence. For instance, 3,150 adolescents who were insured at the time of the survey had an obesity rate of 16.6% (CI: 15.6-17.7%), while 14,481 adolescents had a normal weight prevalence of 61.3% (CI: 60.0-62.5%). Consequently, 172 adolescents without insurance exhibited a slightly lower obesity rate at 15.4% (CI: 11.6-20.0%) even though 67 of them had a notably higher underweight prevalence at 11.5% (CI: 6.2-20.5%). The uninsured group, representing 595 adolescents, also had a lower normal weight prevalence at 57.2% (CI: 49.4-64.7%). The p-value < 0.05 showed significant differences in obesity prevalence across the ACE groups. 

**Table 2:** National outcome measure for socioeconomic variables: percentage of adolescents (age 10-17 years) CI: confidence interval, %: percentage, Pop. Est.: population estimates, (n): number of participants/sample population

Variables	Based on Insurance							Based on childhood experience									
	Insured at the time of survey			Not insured at the time of survey			P-value	No adverse childhood experiences			One adverse childhood experience			Two or more adverse childhood experiences			P-value
Characteristics	%	CI	Sample count	%	CI	Sample count	-	%	CI	Sample count	%	CI	Sample count	%	CI	Sample count	-
Underweight (less than 5th percentile)	7	6.3 - 7.7	1,488	11.5	6.2 - 20.5	67	p-value < 0.05	8.2	7.0 - 9.6	869	7.1	5.6 - 8.9	332	5.6	4.6 - 6.8	334	p-value < 0.05
Normal weight (5th to 84th percentile)	61.3	60.0 - 62.5	14,481	57.2	49.4 - 64.7	595	p-value < 0.05	65	63.2 - 66.8	8,289	56.4	53.6 - 59.2	3,347	56.6	54.1 - 59.1	3,297	p-value < 0.05
Overweight (85th to 94th percentile)	15.1	14.2 - 16.0	3,235	15.9	11.0 - 22.4	152	p-value < 0.05	14.5	13.2 - 15.8	1,687	16.8	14.7 - 19.0	852	15.4	13.7 - 17.2	830	p-value < 0.05
Obese (95th percentile or above)	16.6	15.6 - 17.7	3,150	15.4	11.6 - 20.0	172	p-value < 0.05	12.3	11.1 - 13.7	1,312	19.7	17.4 - 22.3	868	22.4	20.4 - 24.6	1,121	p-value < 0.05

ACEs were another significant factor influencing obesity. A total of 1,121 adolescents with two or more ACEs had the highest obesity rate at 22.4% (CI: 20.4-24.6%), while 868 adolescents with one ACE had an obesity rate of 19.7% (CI: 17.4-22.3%). By contrast, 1,312 adolescents with no ACEs had a lower obesity rate of 12.3% (CI: 11.1-13.7%), even as 8,289 adolescents had a higher normal weight prevalence at 65% (CI: 63.2-66.8%). The p-value < 0.05 showed significant differences in obesity prevalence across the ACE groups. 

## Discussion

The findings of this study reveal significant disparities in obesity rates across different demographic and socioeconomic groups, emphasizing the multifaceted nature of obesity and the profound influence of social determinants on adolescent health outcomes. The rising prevalence of obesity among adolescents is a growing public health concern, and the disparities observed in this study underscore the complexity of addressing this issue effectively. The overall prevalence of obesity observed aligns with recent national estimates. According to the CDC, the obesity rate among United States adolescents aged 12-19 years has stabilized around 20% in recent years, this is comparable to this study's estimated overall obesity prevalence of 21% in adolescents. However, by focusing on the 10-17 age group and stratifying the data by race and socioeconomic factors, this study provides a more nuanced understanding of obesity disparities [12-13].

The analysis reveals pronounced racial disparities in obesity prevalence among adolescents. Hispanic and non-Hispanic Black adolescents exhibit the highest obesity rates, at 21.9% and 21.3%, respectively, compared to 12.7% among their non-Hispanic White peers. This finding is consistent with previous studies, such as Ogden et al. (2014), which reported higher obesity rates among Hispanic and non-Hispanic Black youth, at 22.4% and 20.2%, respectively, compared to non-Hispanic White youth [14]. Furthermore, a recent study conducted by Kim et al. has disclosed comparable findings, indicating that obesity prevalence among Hispanic (25.6%) and non-Hispanic Black (24.2%) individuals aged between two and 19 years was comparatively higher than the obesity prevalence in non-Hispanic Whites (16.1%) and non-Hispanic Asians (8.7%) of similar age group [13]. The persistence of these disparities over time highlights the need for targeted public health interventions that address the specific needs of these communities. Several factors contribute to the higher obesity rates observed among Hispanic and non-Hispanic Black adolescents. Critical factors include socioeconomic status, access to healthy food options, cultural dietary practices, and physical activity levels [14,15]. Hispanic and Black communities often reside in areas with limited access to supermarkets and a higher prevalence of fast food outlets, contributing to poor dietary habits [14,15].

In addition, cultural norms around food and environmental constraints on physical activity exacerbate the risk of obesity in these populations [4,14-15]. The relationship between socioeconomic status and obesity is well-documented, and this study further highlights the significant impact of income and insurance status on obesity rates among adolescents. Adolescents from households with income levels below the FPL show the highest prevalence of obesity, at 22.5%, compared to those from higher-income households (400% FPL or higher), who have an obesity rate of only 9.9%. This inverse relationship between income and obesity is consistent with previous research.

A cross-sectional study by Goto et al. analyzed 21,296 adolescents, revealing that those from low socioeconomic status households had higher obesity rates (22.8%) and were more likely to be non-Hispanic Black (21.7%) [16]. The gap in obesity prevalence between low-income households and others widened significantly over 20 years, with income and education disparities increasing by 1.5 and 1.1 percentage points every four years, respectively [16]. The high prevalence of obesity among low-income adolescents can be attributed to several interrelated factors. Low-income families often face barriers to accessing healthy foods, such as fruits and vegetables, due to their higher cost and limited availability in low-income neighborhoods [10,14]. Financial constraints may limit opportunities for physical activity, as families may not afford extracurricular activities, sports programs, or safe spaces for exercise. The stress associated with financial instability can also contribute to unhealthy eating behaviors and weight gain. Insurance status also plays a crucial role in obesity prevalence. Surprisingly, this study found that uninsured adolescents had a slightly lower obesity rate than insured adolescents (15.4% vs. 16.6%). However, this group also had a higher prevalence of underweight individuals (11.5% vs. 7.0%). This finding diverges from previous research, which generally associates a lack of insurance with higher obesity rates due to limited access to preventive healthcare services. The higher underweight prevalence among uninsured adolescents may reflect underlying issues such as food insecurity or chronic health conditions that are not adequately addressed due to a lack of access to healthcare [14,16-19].

In addition, the data indicate a strong correlation between the number of ACEs and obesity rates. Adolescents with two or more ACEs had the highest obesity rate at 22.4%, compared to those with no ACEs, who had an obesity rate of 12.3%. This finding aligns with the broader literature, which has established a link between ACEs and a range of negative health outcomes, including obesity. A study by Offer et al. (2022) has highlighted the relationship between childhood trauma and obesity, showing that individuals who experienced multiple ACEs were more likely to engage in unhealthy behaviors, such as overeating, as a coping mechanism for emotional distress [20]. The mechanisms underlying the association between ACEs and obesity are complex and multifactorial. Chronic stress resulting from ACEs can lead to dysregulation of the hypothalamic-pituitary-adrenal (HPA) axis, resulting in elevated cortisol levels that promote fat accumulation, particularly in the abdominal region [21]. ACEs are also associated with behavioral health issues, including depression and anxiety, which can contribute to emotional eating and weight gain. The environments in which children with ACEs grow up may lack stability and support, further compounding the risk of obesity through poor diet and lack of physical activity [22-23].

The study's findings show the importance of developing and implementing targeted interventions that consider the unique challenges faced by these communities, such as improving access to healthy foods, creating safe spaces for physical activity, and providing support for families experiencing ACEs. Addressing the multifaceted nature of adolescent obesity will require a comprehensive approach that includes public health initiatives, community-based interventions, and policy changes to reduce disparities and promote healthier outcomes for all adolescents. This study utilizes a large, nationally representative dataset, allowing for a robust analysis of obesity prevalence among diverse racial and socioeconomic groups. The stratification by income, insurance status, and adverse childhood experiences provides a comprehensive understanding of the multifaceted factors contributing to adolescent obesity. Despite its strengths, the study relies on self-reported data, which may be subject to bias or inaccuracies. In addition, the cross-sectional design limits causal inferences, making it difficult to establish temporal relationships between obesity and the examined variables.

Limitations of the study

Among the notable limitations of this study include the observation that the cross-sectional nature of data drawn from NSCH, which does not offer the necessary insight into causes and causality: aspects like income might not correlate with the socioeconomic status of the adolescents’ households. Therefore, this necessitates longitudinal design use in the establishment of temporal precedence, along with the inclusion of other behavioral, biological, and environmental measurements. Still, despite the observation that BMI was mainly based on objective measurements, reporting errors might have existed, including the confounding measurement units. This is a widespread challenge with BMI datasets that are longitudinal. However, for this study, the necessary steps were undertaken to ascertain that the longitudinal datasets used are robust.

Public health implications and recommendations

The findings of this study have important implications for public health policy and practice. First, there is a clear need for targeted interventions that address the specific needs of minority and low-income adolescents. In this regard, the medical field is tasked with addressing the high obesity prevalence in adolescents through the integration of trauma-informed care, acknowledging the association between obesity and ACEs, and offering holistic support via social and mental health services. Thus, there is a need for physicians to advocate for enhanced access to care, particularly for minority and low-income households adolescents through support for expansion of insurance coverage and access to effective preventive services. In addition, the multidisciplinary approach that involves psychologists, social workers, and nutritionists is vital to tackling the intricate obesity factors. Moreover, healthcare professionals should drive changes to public policies to effectively address the observed socioeconomic disparities while also promoting healthy environments for adolescents.

Second, the strong association between ACEs and obesity highlights the need for a trauma-informed approach to obesity prevention. Public health programs should incorporate screening for ACEs and provide appropriate support and resources for adolescents and their families to address the underlying trauma that may contribute to unhealthy behaviors. Finally, ensuring all adolescents' healthcare access is crucial for effective obesity prevention and management. Expanding insurance coverage and ensuring that preventive services, such as nutritional counseling and weight management programs, are accessible to all adolescents, regardless of income, could help reduce obesity rates and improve overall health outcomes.

## Conclusions

This study provides valuable insights into the complex interplay between race, socioeconomic factors, and obesity among United States adolescents. The significant disparities observed in obesity prevalence highlight the need for comprehensive public health strategies that address the social determinants of health. By focusing on the unique needs of vulnerable populations, expanding access to healthcare, and addressing the underlying causes of childhood adversity, we can work toward reducing obesity rates and improving the overall health and well-being of adolescents across the nation. Future research should continue to explore these relationships and identify effective interventions to combat adolescent obesity.
